# iNAP: An integrated network analysis pipeline for microbiome studies

**DOI:** 10.1002/imt2.13

**Published:** 2022-03-16

**Authors:** Kai Feng, Xi Peng, Zheng Zhang, Songsong Gu, Qing He, Wenli Shen, Zhujun Wang, Danrui Wang, Qiulong Hu, Yan Li, Shang Wang, Ye Deng

**Affiliations:** ^1^ CAS Key Laboratory of Environmental Biotechnology, Research Center for Eco‐Environmental Sciences Chinese Academy of Sciences Beijing China; ^2^ Collegeof Resources and Environment University of Chinese Academy of Sciences Beijing China; ^3^ Institute for Marine Science and Technology Shandong University Qingdao China; ^4^ College of Horticulture Hunan Agricultural University Changsha China; ^5^ West China Hospital of Stomatology, State Key Laboratory of Oral Diseases, National Clinical Research Center for Oral Diseases Sichuan University Chengdu China

**Keywords:** interaction, microbial association, microbiome, network analyses

## Abstract

Integrated network analysis pipeline (iNAP) is an online analysis pipeline for generating and analyzing comprehensive ecological networks in microbiome studies. It is implemented in two sections, that is, network construction and network analysis, and integrates many open‐access tools. Network construction contains multiple feasible alternatives, including correlation‐based approaches (Pearson's correlation and Spearman's rank correlation along with random matrix theory, and sparse correlations for compositional data) and conditional dependence‐based methods (extended local similarity analysis and sparse inverse covariance estimation for ecological association inference), while network analysis provides topological structures at different levels and the potential effects of environmental factors on network structures. Considering the full workflow, from microbiome data set to network result, iNAP contains the molecular ecological network analysis pipeline and interdomain ecological network analysis pipeline (IDENAP), which correspond to the intradomain and interdomain associations of microbial species at multiple taxonomic levels. Here, we describe the detailed workflow by taking IDENAP as an example and show the comprehensive steps to assist researchers to conduct the relevant analyses using their own data sets. Afterwards, some auxiliary tools facilitating the pipeline are introduced to effectively aid in the switch from local analysis to online operations. Therefore, iNAP, as an easy‐to‐use platform that provides multiple network‐associated tools and approaches, can enable researchers to better understand the organization of microbial communities. iNAP is available at http://mem.rcees.ac.cn:8081 with free registration.

## Highlights

- Integrated network analysis pipeline (iNAP) contains two network analyses pipelines for intradomain and interdomain associations of microbial species at multiple taxonomic levels, that is, molecular ecological network analysis pipeline and interdomain ecological network analysis pipeline.
- iNAP provides multiple approaches and methods for network analysis of microbiome studies, including SparCC, eLSA, SPIEC‐EASI, and RMT‐based Pearson's or Spearman's correlations.
- Through free registration and easy operations, iNAP can be easily operated by researchers without any prerequisite bioinformatics or statistical language‐based programming skills for network analyses of microbiome studies.

## Graphical abstract

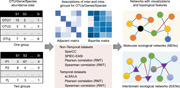

## INTRODUCTION

Elucidating the complex relationships of microbial dark matter, such as microbial interactions, from massive metagenomics sequencing data provides the possibility to better understand the assembly mechanisms of microbial communities in free‐living and host‐associated habitats [1]. Microbial network analysis is a popular approach to explore microbiome metagenomic data sets and find insightful perspectives in complex ecosystems, such as soils [2], and at different scales. Network analysis can be used to detect disproportionally important species or keystones within communities that may help systems resist external disturbances or assist in the invasion of hosts [3]. Moreover, at the community level, the structure of networks may indicate the robustness or stability of ecosystem services in response to environmental disturbance [4] or species loss [5]. Not only intradomain species relationships but also the interdomain species associations in microbial communities, for example, the hierarchal structure of multiple trophic microorganisms, have been drawing substantial attention [6]. Even though a number of challenges regarding the use of network analysis for microbial ecology studies have been raised [7], with cautious usage and meaningful interpretations, network analysis can still contribute greatly to ecological research [2].

However, due to the physical scale of microorganisms, microbial network analysis based on microbiome data sets depends on various statistical methods and newly developed approaches. For Pearson's or Spearman's correlation‐based methods, molecular ecological network analyses (MENA) [8] identify a threshold based on random matrix theory (RMT) to filter fewer but still robust microbial associations [9], which can be conducted automatically. Sparse correlations for compositional data (SparCC) [10] and sparse inverse covariance estimation for ecological association inference (SPIEC‐EASI) [11] also perform well to infer microbial associations and provide python‐based script and R packages, respectively. Extended local similarity analysis (eLSA) [12] and liquid association (LA) [13] explore time‐dependent microbial associations for time series data. In addition, more detailed methods for network inference and strategies for a trade‐off between methods and complexity have been summarized to help better characterize microbial communities [14]. Thus, considering the various methods and tools for network analysis, we developed the integrated network analysis pipeline (iNAP) to implement various network construction methods and topological feature analyses for both intradomain [8] and interdomain ecological networks [15] for microbiome data sets. iNAP also provides statistical approaches to explore the effects of abiotic factors on network structures, which also contribute to the microbial assembly processes. With many versatile functions relevant to network analysis, this protocol mainly describes the fundamental workflow of iNAP and the contained analysis tools in a comprehensive manner.

## PIPELINE OVERVIEW AND IMPLEMENTATIONS

**Figure 1:**
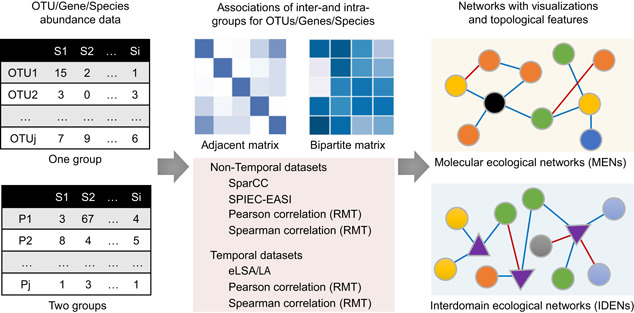
Simplified schematic plot to show the tools sections in iNAP for network analysis. eLSA, extended local similarity analysis; iNAP, integrated network analysis pipeline; LA, liquid association; RMT, random matrix theory; SparCC, sparse correlations for compositional data

Our proposed iNAP pipeline integrates the molecular ecological networks (MENs) [8] and interdomain ecological networks (IDENs) [15] from microbiome abundance data, for example, species (genes/OTUs) abundance data set (Figure 1). Based on the metagenomic abundances for species/genes, the intergroup and intragroup associations between species or genes can be obtained using various statistical approaches. For nontemporal data sets, SparCC, SPIEC‐EASI, and RMT‐based Pearson's and Spearman's could be alternative options, while for temporal data sets, eLSA/LA and RMT‐based Pearson's and Spearman's approaches might be candidate methods to generate network matrices. We used the adjacent network matrix and bipartite network matrix to show the relationships for intradomain and interdomain groups of species. Accordingly,  MENs and IDENs show the potential associations of species within complex microbial communities, and the topological network structures could reflect some potential ecological implications, for example, modular structure to indicate similar functions [8]. iNAP provides various comparably feasible alternatives for researchers to construct networks based on metagenomics abundance data.

**Figure 2:**
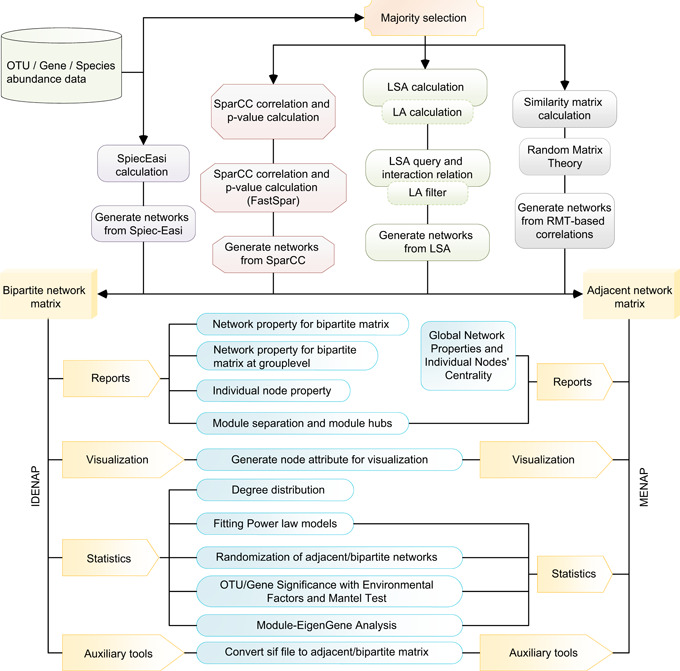
The workflow and tools to exhibit the steps of network construction and analysis in iNAP. iNAP, integrated network analysis pipeline; LA, liquid association; SparCC, sparse correlations for compositional data

Implemented within the Galaxy project framework [16], iNAP can be easily operated by researchers without any prerequisite bioinformatics or statistical language‐based programming skills. Moreover, iNAP provides various network approaches and statistical analysis methods for topological features (Figure 2). From the beginning, with microbial metagenomic abundances (Box 1), everyone can easily follow this detailed workflow to conduct network analysis and obtain relevant interpretation results. Due to the multiple alternative options for network construction, this protocol mainly describes the network construction and analysis for IDENs as an example; for the analysis of MENs, we instead refer to the steps that are similar in IDENs.

Box 1 **Data set formats for input files**

This box describes the data formatting of input files for processing in iNAP. Generally, the pipeline accepts files with tab‐separated text with the file extension of txt or tabular. For the headers or row names of the input file (usually the sample names or species names), we recommend the use of alphabet letters as the first character, with a combination of numbers, rather than a number, for example, S1, P1, and OTU1, instead of 1S, 1P, and 1OTU. The underscore character should be used to replace any special symbols, including blank, asterisk, and hashtag, to prevent potential problems. Since iNAP uses “_M” and “_P” to distinguish the two groups of datasets, please avoid using these labels in species names. The formats can be referenced in the example dataset in “shared libraries” of iNAP.

**Abundance data sets (.txt/.tabular)**

The abundance data set contains the species abundance across different samples. The data set files are formatted for species abundance in rows and samples in columns. The header line can be started with “OTUID,” “OTU,” or “tabular symbol.”

**Metadata files (.txt/.tabular)**

The metadata file usually includes the environmental factors associated with the samples of the abundance data set. The sample names are in rows and environmental factors are in columns in this file. The order of the sample names should be consistent with the abundance data sets.

**Taxonomy files (.txt/.tabular)**

The taxonomy file contains the detailed taxonomic information of the species IDs shown in the abundance data sets. Normally, the file should contain several columns with domain, phylum, class, order, family, genus, and species to indicate the finer taxonomic information of species.

### Procedures for network constructions

Currently, iNAP provides four approaches for the calculation of potential associations between species and generates network results. Herein, we describe the protocols separately with different methods, taking the interdomain ecological network analysis pipeline (IDENAP) as an example to show the primary steps.

#### Method 1: SparCC

1. *Majority selection*. Considering the sparsity of microbiome data sets, we recommend keeping the prevailing species across various samples, for example, minimum of half of the total samples in default. For IDENAP, two input data set files (e.g., microbial abundance data set and plant abundance data sets) should be added here and the majority for each data set can be adjusted accordingly. Currently, iNAP requires a minimum of eight samples as replicates for network construction. After this step, the remaining data set should contain less than 1000 species due to the large computational resources required for most approaches. Otherwise, the following steps would result in error messages such as “Our pipeline cannot process the dataset with larger than 1000 OTUs/genes for this method.” For larger data sets, we suggest running the association calculations on local computation machines accordingly; these steps are available in Github (https://github.com/yedeng-lab/iNAP).
2. *SparCC correlation and p value calculation*. The filtered table from Step 1 is selected as the input file for this step. By using the default parameters to calculate the correlation values of SparCC [10], including 20 as the number of inference iterations, 10 for exclusion iterations, 0.1 as strength exclusion threshold, and 100 as the number of times shuffled, two output files containing correlations and a pseudo *p* values matrix are exported. Essentially, the large number of shuffling consume a great amount of computation time and 100 shuffles, as a default option, could deal with most cases. In addition, we also implemented the FastSpar method to calculate these outputs for the SparCC method, which is an efficient alternative way to handle larger data sets (more than 1000 species) [17].
3. *Generate networks from SparCC*. With a self‐determined threshold and different significance levels possible, only correlations larger than the threshold with associated pseudo *p* values that are less than the chosen significance level would be retained to generate the network matrix file. For example, the default value of SparCC would be 0.3 as the threshold value and 0.05 as the significance level. Since there were two data sets of species in Step 1, the network matrix should be in bipartite network mode; otherwise, an adjacent network for only one group of species would be generated (Details in Box 2). Moreover, the output format can be adjusted accordingly, with different visualization approaches, that is, Gephi or Cytoscape.

Box 2 **Descriptions for output files**

After the network construction using the selected methods (Figure 2), one network matrix, either adjacent or bipartite, is exported and the associated files are simultaneously generated for visualization. For IDENAP, there are four kinds of network results, that is, one whole network, one bipartite network between two groups, and two adjacent networks for each of the species groups. For MENAP, there is only one kind of network exported, that is, the adjacent network matrix.

**Adjacent network matrix**

The adjacent network matrix is a square matrix containing the same ordered row and column names, and the matrix is adjacent with a diagonal position of zero values. The matrix is 0/1 mode to show the absence/presence of the potential associations between pairwise species.

**Bipartite network matrix**

Generally, the bipartite network matrix contains two groups of species and one level of species in columns and another level of species in rows. There are no intersections between column and row names. The 0/1 mode is used within the matrix to show the absence/presence of potential associations.

**Figure 3:**
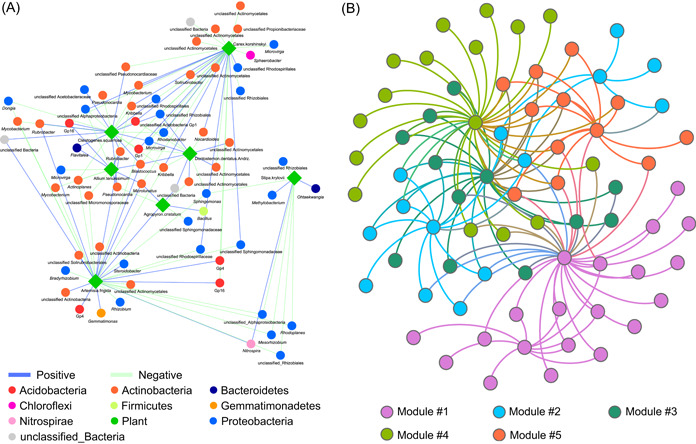
Visualization of networks from the iNAP data using Cytoscape (A) and Gephi (B). The nodes were colored with different taxonomic groups (A) and their module compartments (B). iNAP, integrated network analysis pipeline

For the visualization of networks, the output of iNAP can be directly imported to two public‐friendly software programs, Cytoscape [18] and Gephi [19], both of which can be exported (Figure 3).

**Files for Cytoscape**

Three files are provided by iNAP: one file (.sif), one node attribute file (.txt), and one edge attribute file (.txt). The  .sif file can be imported to Cytoscape software directly. The node attribute file contains the specific node features in the network and the edge attribute file showed the positive/negative association between nodes and the corresponding coefficient values. Files formatted in a manner similar to the three files above can be imported as well.

**File for Gephi**

Two files are provided in iNAP: one node attribute file (.csv) and one edge attribute file (.csv). The edge attribute file can be imported to Gephi software as an edge feature and the node attribute file can be imported accordingly to show the node features in the network. Similarly, formatted files can be imported as well.

#### Method 2: SPIEC‐EASI

4. *SpiecEasi calculation*. The parameters related to majority selection can be found in Step 1. The parameters for SPIEC‐EASI all have default options [11], including glasso as the estimation method, 20 as the penalties (possible range of 10–100) changing with a ratio of 0.001 from the minimum value to theoretic maximum value for penalty parameter, and 0.05 as the threshold for Stability Approach to Regularization Selection (StARS) criterion. The number of subsamples can be adjusted according to the samples of the input data set, and it should be noted that large values take a long time. The three output files are the SpiecEasi matrix with inverse covariance estimation results, SpiecEasi report containing parameters during the computation and estimations, and the filtered matrix after the majority selection.
5. *Generate networks from SpiecEasi*. Based on the SpiecEasi matrix from Step 4, the network matrix and associated input files for visualizations are generated (see details in Box 2).

#### Method 3: eLSA

6. *Majority selection*. Operations like Step 1 (see above descriptions).
7. *LSA calculation*. The input file is the filtered table from Step 6. Since the LSA method deals with temporal data sets, sample columns of the input files should be ordered according to time points and that the replicates for each time point are evenly distributed. The maximum time delay can be adjusted with the time points. Other parameter options are default from the LSA method [12], including permutation to estimated *p* value with a precision of 0.001. Bootstrap of 100 times is recommended for nonreplicated data. Zero would be filled in as missing data and averaging is used to summarize the replicated data. The output of this step is a collapsed table for each pairwise association between species, containing LS values and the associated *p* and *Q* values.
8. *LSA query and interaction relation*. After obtaining the LS values of pairwise associations between species from Step 7, this step aims to filter the LS values with certain criteria, for example, absolute value of LS larger than 0.28, *p* < 0.001, and *Q* < 0.05. There is “Insert Additional Query Condition” for more query conditions, and each query condition indicates one query formula. Lower filter query conditions might work if this step results in an error message. The output file is a subset of the data set of Step 7.
9. *(optional) LA calculation and LA filter*. LA scores are developed to calculate the pairwise association depending on a third mediator species. This step is optional for normal LSA workflow. The parameters related to this step are Step 7 and Step 8.
10. *Generate networks from LSA*. Based on the filtered LSA pairwise results from Step 8, the network results would be exported accordingly, with two options for visualization approaches and two potential network modes (adjacent and bipartite networks). See details in Box 2.

#### Method 4: RMT‐based correlations

11. *Majority selection*. Similar operations to Step 1 (see above descriptions).
12. *Similarity matrix calculation*. Pearson's and Spearman's correlations methods are provided to calculate the pairwise associations. Before calculation, the empty values or zero values would be suggested to be filled with a small value (e.g., 0.01) for paired valid positions. Alternatively, the options of keeping blank or zero, and filling all with smaller values are also provided. Taking the logarithm or not is also another pretreatment for microbiome data. If the data set is temporally dynamic, a sample description file to show the replicates and time points is required as well. Details are given on the help page of iNAP. The output file contains an adjacent correlation coefficient matrix and the corresponding *p* values.
13. *Random Matrix Theory*. The adjacent correlation matrix from Step 12 can be imported to this step. In this step, the correlation matrix would be scanned with the cutoff set from 0.01 to 1, with a step of 0.01, to find the potential transition point of network eigenvalues from Poisson distribution to Gaussian orthogonal ensemble statistics. The output matrix lists the results of cutoff and corresponding goodness‐of‐fit using *χ* ^2^. It is suggested that the recommended cutoff is the value at which the statistics of *χ* ^2^ rejects the hypothesis. Another output file shows the cutoff distributions at significance levels of 0.05 and 0.001, and the candidate cutoffs are above significance lines.
14. *Generate networks from RMT‐based correlations*. Using the selected cutoff based on the RMT of Step 13, the network output files would be exported accordingly containing the correlation values and associated *p* values. Also, the two visualization approaches and two network modes are provided (see details in Box 2). For time series data, there is an additional matrix file required from Step 12.

### Procedures for network analysis

15. *Network property for bipartite matrix*. The input file for this is the bipartite network matrix (Box 2). For the absence/presence mode of the network matrix, the network should be unweighted. The output file consists of topological features of the bipartite network, such as nested structure and compartments. Details of the results index are summarized in the help page for this tool in iNAP.
16. *Network property for bipartite matrix at group level*. The input file for this is the bipartite network matrix (Box 2). It should be in unweighted mode since the matrix in iNAP is an absence/presence pattern. The result of this step is most similar to Step 15 and the detailed indexes are summarized in the help page for this tool in iNAP.
17. *Individual node property*. The input file for this is the bipartite network matrix (Box 2). The results include various properties for each species of the network at the node level, including species degree to show the links to this node, and betweenness and closeness to describe the centrality of the node in the network. Detailed explanations are provided on the help page of this tool.
18. *Module separation and module hubs*. The input file for this is the bipartite network matrix (Box 2). For the module detection methods, iNAP provides four popular methods, including greedy modularity optimization [20], short random walks [21], leading eigenvector [22], and simulated annealing [23]. In general, simulated annealing may obtain good performance for bipartite networks. The output text file shows the modularity of the bipartite network and the module orders for each species. The within‐module connectivity (*Z*) and among‐module connectivity (*P*) for each species are exported as well. With certain assignment criteria [24], the species could be classified into network hubs, module hubs, connectors, and peripherals. The associated *Z*–*P* plot is also exported in this step.
19. *Generate node attribute for visualization*. By importing the two files from Step 17 and Step 18, this step combines the results and exports a formatted node property file, which can be used for Cytoscape and Gephi visualization (Figure 3).
20. *Degree distribution*. The input file for this is the bipartite network matrix (Box 2). The output for this step contains the degree distributions (cumulative distribution vs. number of links) for each group of the network. Three fitting models are used to indicate the goodness‐of‐fit, including exponential, power law, and truncated power law.
21. *Fitting power law models*. The input file for this is the bipartite network matrix (Box 2). This step is like Step 20 with minor differences as it will use power law, log power law, exponential law, and truncated power law to fit the degree distributions.
22. *Randomization of bipartite networks*. The input file for this is the bipartite network matrix (Box 2). Since this step calculates the network properties for random network matrices, the parameters are consistent with Step 15. Three random network generation approaches are provided, that is, rewiring links by keeping node degree constant, shuffle.web, and mgen. The number of random matrices can be adjusted as well. The output contains the average value and standard deviations for each network topological property based on hundreds of random networks. In addition, the detailed result for each random network is also exported.
23. *OTU/Gene significance with environmental factors and mantel test*. The first step is to calculate the correlation coefficient between species within the network and environmental factors, based on the three input files, that is, the filtered matrix from Step 1, bipartite network matrix (Box 2), and an uploaded environmental factor file (Box 1). Pearson's and Spearman's methods are included and the standardized method and treatment of missing values can be adjusted as well. After obtaining the correlation result, based on the degree property for each node in the network from Step 17, Mantel and partial Mantel tests are used to evaluate the relationships between network topological structure and environmental factors. The result file indicates the Mantel *r* value and *p* value to show the significance.
24. *Module‐EigenGene Analysis*. Three input files, that is, the filtered matrix from Step 1, an uploaded environmental factor file (Box 1), and modularity results from Step 18 are required. Modules with a small number of members (e.g., five members) would be ignored. The output file is a formatted web page that contains result tables and plots to indicate the module‐eigengene for each module, hierarchy structure, and modules correlations to environmental factors.

### Procedures for auxiliary tools

25. *Convert sif file to bipartite matrix*. This step aims to generate a bipartite matrix from a sif file exported from Cytoscape software. The two groups of species can be separated according to the provided unique label, for example, “B_OTU” and “P_OTU.” The output can be used for bipartite network matrix analysis (Figure 2).
26. *Taxonomy summary of low‐level species for bipartite networks*. The input files for this step are the bipartite network matrix (Box 2) and species taxonomy file (Box 1) to count the species at higher taxonomic levels (e.g., phylum or class) and generate subbipartite networks. The output files include a summarized table and associated plot to show the abundance for each subnetwork, and a zipped file of bipartite network matrix at the selected taxonomic level.

### Brief steps for adjacent networks

For the molecular ecological network analyses pipeline (MENAP, Figure 2), due to the adjacent network consisting of a single group, the parameters during network construction should shift to “one group of species” in “majority selection” step. The following steps should take care of this accordingly. After network generation, there is one adjacent matrix to show the potential interactions between all species with presence/absence data and the computations of further topological features.

27. *Global network properties and individual nodes' centrality*. The input for this step is an adjacent network matrix (Box 2). The output files include one table containing various network topological features for global networks, for example, average degree, cluster coefficient, and average path distance, and another table measuring node features. Details are given on the help page for this tool.
28. *Module separation and module hubs*. The input for this step is an adjacent network matrix (Box 2). There are four module classification methods in iNAP (see details in Step 18).
29. *Generate node attribute for visualization*. Importing the results from Step 27 and Step 28, the node attribute file can be generated for Cytoscape or Gephi software to indicate the node features in networks (Figure 3).
30. *Fitting power law models*. The input file for this step is an adjacent network matrix (Box 2). The output file contains the four fitting models for degree distributions, including power law, log power law, exponential law, and truncated power law.
31. *Randomization of adjacent networks*. The input file for this step is an adjacent network matrix (Box 2). Consistent with Step 28 for modularity calculation, 100 random networks are generated by rewiring the links from the imported matrix and then the summarized random network features are exported. The nonrandomness of topological features between the random network matrix and the empirical network matrix of Step 27 can be compared using Student's *t* test.
32. *OTU/Gene significance with environmental factors and Mantel test*. By importing the filtered majority table from Step 1, modularity result from Step 28 and the uploaded environmental factors (Box 1), correlations between networked species and environmental factors can be obtained and further Mantel tests can be used for linking the correlations to network topological structure, mainly node connectivity from Step 27. The output shows the correlation coefficient of the Mantel test and the associated significance level.
33. *Module‐EigenGene Analysis*. After importing the filtered majority table from Step 1, modularity result from Step 28, and the uploaded environmental factors (Box 1), module‐eigengene analysis can be conducted by ignoring small modules, those with less than five members, in default settings.
34. *Convert sif file to an adjacent matrix*. Any exported sif file from Cytoscape software can be imported here and converted to an adjacent network matrix for network analysis from Step 27 to Step 33.

### iNAP features and applications

iNAP is designed and maintained in a SAFE mode, that is, stability, accessibility, feasibility, and easiness. The pipeline would be maintained by stable technical support to ensure public accessibility. With different steps in the workflow and auxiliary tools (Figure 2), users can upload their own data sets and temporary files during local computations for network files into iNAP and follow the subsequent steps. Moreover, the users can use tools crossly in iNAP, for example, RMT method can be used for the SparCC correlation matrix to determine the cutoff value. By implementing multiple network construction methods and a Galaxy framework, it is convenient for researchers following this protocol to obtain their network results from microbiome data sets, and with the help of iNAP, it is possible for microbial ecologists to explore the assembly mechanisms of microbial communities and decipher how the stability of ecosystems is mediated by keystone organisms and ecosystem functions [3, 6, 25].

### Troubleshooting

The advice for troubleshooting is summarized in Table 1.

**Table 1:** Troubleshooting table

Step	Problem	Possible reason	Solutions
All	Tool does not start to run	Too many programs running within the account or in the server	Wait till the program finishes or stop the previous program
	Error messages	Selected wrong input file	Check with the file format with reference file in Box 1 and example files in the pipeline. Follow the protocol steps accordingly
1	Empty results	High majority	Decrease the majority
2, 4, 7, 12	Large species numbers	Too many species	Decrease the species numbers for the import file or run the program locally
	Less sample numbers	Sample numbers less than eight	Increase the number of replicates for the samples
3, 5, 10, 14	Empty results	No result for the provided selection criterion	Downscale selection criterion, for example, lower threshold
23, 24, 32, 33	Empty results	Inconsistent sample names in the input file	Manually check the sample names for the input files and name formats in Box 1

## CONFLICTS OF INTEREST

The authors declare no conflicts of interest.

## AUTHOR CONTRIBUTIONS

All authors contributed to the pipeline development and workflow of analyses. The initial idea and framework were conceived by Kai Feng and Ye Deng. The pipeline was built and maintained by Kai Feng and Xi Peng. During the pipeline development, Zheng Zhang, Songsong Gu, Qing He, Wenli Shen, Zhujun Wang, Danrui Wang, Qiulong Hu, Yan Li, and Shang Wang contributed to the pipeline test and usage for network analyses. This protocol was written by Kai Feng and Ye Deng, and revised with from other authors' suggestions.

## Data Availability

iNAP is publicly accessible to all researchers (https://mem.rcees.ac.cn:8081) and users can register themselves without any permissions or restrictions. All analysis tools can be used by anonymous users, but we encourage researchers to conduct network analyses with a registered user account to ease pipeline management. Moreover, suggestions for computations of SparCC, LSA, and SPIEC‐EASI in local computation machines are deposited in Github (https://github.com/yedeng-lab/iNAP).
